# Reproducing indolent B-cell lymphoma transformation with T-cell immunosuppression in LMP1/CD40-expressing mice

**DOI:** 10.1038/s41423-018-0197-6

**Published:** 2019-01-11

**Authors:** Christelle Vincent-Fabert, Alexis Saintamand, Amandine David, Mehdi Alizadeh, François Boyer, Nicolas Arnaud, Ursula Zimber-Strobl, Jean Feuillard, Nathalie Faumont

**Affiliations:** 1 CNRS-UMR 7276 INSERM U1262 CRIBL, University of Limoges, and Hematology Laboratory of Dupuytren Hospital University Center (CHU) of Limoges, Limoges, France; 2 INSERM U917, University of Rennes 1, Rennes, France; 3 LNPRM, EFS Bretagne, Rennes, France; 4 Research Unit Gene Vectors, Helmholtz Center Munich, German Research Center for Environmental Health GmbH, Munich, Germany

**Keywords:** B-cell lymphoma, Immunosurveillance

Indolent B-cell lymphomas are a group of incurable cancers of the elderly that encompass various entities, such as chronic lymphocytic leukemia (CLL), follicular lymphoma (FL), and marginal zone lymphoma (MZL). All indolent lymphomas may evolve towards aggressive transformation with an increased proliferation index and decreased tumor doubling time. This transformation, which is called Richter’s syndrome in CLL, is associated with an aggressive clinical course and poor survival. At least in CLL, transformation of an indolent B-cell clone is molecularly different from that of de novo diffuse large B-cell lymphomas.^1^ Aging is known to be associated with immune decline, with increased inflammation, decreased immune surveillance and increased onset of malignancies among the consequences.^2^ Progression of indolent B-cell lymphomas is likely to be associated with escape from immune surveillance.^3^ Therapies targeting the PD-1/PD-L1 axis seem to be effective only for CLL with Richter’s transformation.^4^ However, the role of the immune system in this transformation process is poorly understood.

Our aim was to experimentally address the role of immune suppression in the transformation of an indolent B-cell lymphoma. Among the very few mouse models of indolent lymphoma, latent membrane protein 1 (LMP1)/CD40 chimeric protein-expressing mice (LMP1/CD40-expressing mice) are characterized by B cell-specific continuous CD40 signaling, which is responsible for spleen indolent clonal or oligoclonal B-cell lymphoma after one year in 60% of cases.^5^

**Fig. 1:**
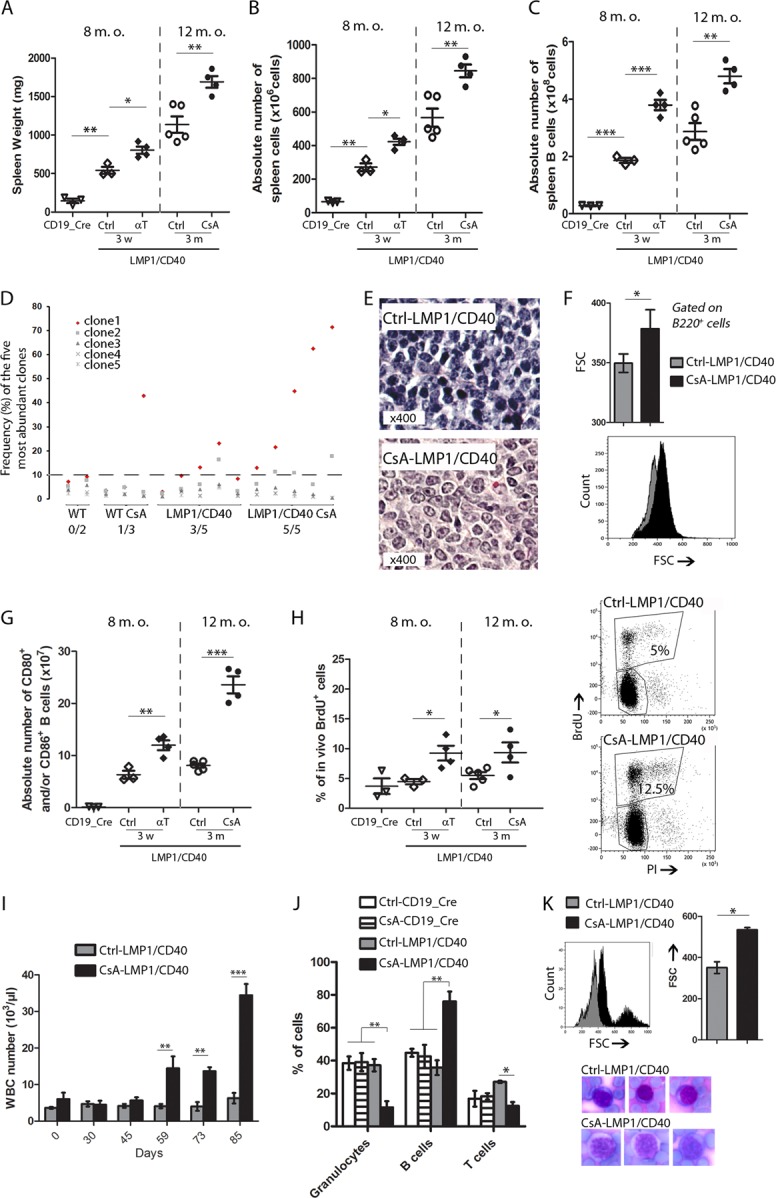
Immune suppression induces transformation of LMP1/CD40 indolent B-cell lymphomas: To induce antibody-mediated T-cell depletion, LMP1/CD40-expressing mice were injected intraperitoneally every 4 days for three weeks with a mix of anti-CD4 (YTS 191.1.2), anti-CD8 (YTS 169.4.2.1), and anti-Thy-1 (YTS 154.7.7.10) antibodies in In VivoPure Dilution Buffer at a dose of 200 µg each (Bio X Cell; USA). For CsA treatment, wild type and LMP1/CD40-expressing mice were injected intraperitoneally daily for three months with placebo or 10 mg/kg of CsA (Sandimmun – Novartis; USA) diluted in 5% glucose. **a** Means and standard deviations of the spleen weights from the wild type (CD19_Cre) or LMP1/CD40-expressing mice immunosuppressed (αT and CsA) or not (Ctrl). **b** Absolute numbers of spleen cells per spleen. **c** Absolute numbers of B lymphocytes per spleen. **d** Frequency of cases with a clonal abundance greater than 10%. The clonal abundance was estimated after high-throughput sequencing of the VDJ regions. Transcripts were amplified by 5’RACE PCR using a reverse primer that hybridized within the µ CH1 exon as previously described.^6^ The amplicons were sequenced on an Illumina MiSeq sequencing system using the MiSeq kit Reagent V2 500 cycles. Repertoire analysis was performed using the IMGT/HighV-QUEST tool and the R software. Briefly, the VH, JH, and CDR3 segments were identified using HighV-QUEST. Based on these annotations, the reads were grouped into clonotypes that shared the same VH and JH genes and high CDR3 homology. Then, the relative abundance of each clonotype was calculated. **e** Hematoxylin and eosin staining of spleen sections from control LMP1/CD40-expressing mice (upper panel) or mice immunosuppressed with cyclosporine A (CsA) (lower panel). **f** Flow cytometry estimation of the spleen B-cell sizes from the Ctrl and CsA-LMP1/CD40-expressing mice. Upper panel, means and standard deviations of the forward scatter (FSC) for all studied mice. Lower panel, representative overlay of the FSC monoparametric histograms gated on B220^+^ B cells. **g** Means and standard deviations of the flow cytometry percentages of B220-positive spleen B cells expressing the CD80 and/or CD86 activation markers. **h** Right panel: Means and standard deviations of flow cytometry percentages of BrdU-positive B cells after in vivo BrdU incorporation. Left panel: Representative biparametric histogram showing the intensity of propidium iodide (PI, *x* axis) and BrdU (*y* axis) staining for the control and CsA-treated LMP1/CD40-expressing mice. The percentage of proliferating cells is indicated in the graph. For the in vivo proliferation assay, the mice were injected intraperitoneally with 2 mg of BrdU 18 h prior to cell isolation. The splenocytes were stained for B220, and the cell cycle phases were analyzed using the FITC-BrdU Flow Kit (BD Pharmingen). **i** Numbers of circulating white blood cells in the LMP1/CD40-expressing mice immunosuppressed (CsA) or not (Ctrl). **j** Flow cytometry percentages of circulating granulocytes (Gr-1^+^), B cells (B220^+^) and T cells (CD3^+^). **k** Upper panels: Flow cytometric estimation of circulating B-cell sizes from the Ctrl and CsA- LMP1/CD40-expressing mice. Overlay of FSC monoparametric histograms gated on B220 B cells (left panel). Means and standard deviations of the FSC for all studied mice (right panel). Lower panel: Representative lymphocyte morphology after May-Grünwald Giemsa staining of blood smears from the Ctrl and IS mice (magnification × 1000). For all experiments, at least four mice were studied for each condition. Significant differences are indicated by * (*p* < 0.05), ** (*p* < 0.01) and *** (*p* < 0.001)

Eight-month-old LMP1/CD40-expressing mice were treated either every 4 days with a cocktail of monoclonal antibodies (mAbs) against T and NK cells for 3 weeks or daily with Cyclosporine A (CsA, one of the main immune suppressive drugs used in organ transplantation) for 3 weeks or 3 months. T-cell depletion and CsA treatment had no effect on the spleen size and weight in the CD19-Cre mice (not shown). After three weeks of treatment with mAbs against T and NK cells, T-cell depletion was very pronounced in the spleen and was almost complete in the blood (not shown). After CsA treatment, T cells were strongly decreased in the blood but only mildly decreased in the spleen (not shown). T- and NK-cell depletion by mAbs was associated with increased spleen enlargement (Fig. 1a).^6^ This spleen enlargement was related to increased splenocyte absolute numbers (Fig. 1b), which was mainly due to B-cell compartment expansion (Fig. 1c). Three weeks of immunosuppression with CsA had no significant effect on spleen size (not shown). After 3 months, the spleens of the control LMP1/CD40-expressing mice were further enlarged due to aging (Fig. 1a). However, three months of immunosuppression with CsA induced more pronounced splenomegaly (Fig. 1a) with a significant increase in the absolute numbers of spleen cells (Fig. 1b) that was also due to an increase in the splenic B-cell content (Fig. 1c).

B-cell clonality was analyzed by high-throughput sequencing of the VDJ regions (Fig. 1d). With a threshold of a 10% clonal frequency (or clonal abundance), no significant clonal expansion was seen in the one-year-old control wild type mice. Although the spleen morphology remained globally unchanged without splenomegaly and B-cell expansion, three months of CsA treatment in the control mice was associated with the presence of spleen B-cell clones with a rate of greater than 10% in one out of the three tested cases (33%) cases. Three out of the five (60%) untreated LMP1/CD40-expressing mice exhibited clonal B-cell expansion. Five out of five (100%) LMP1/CD40-expressing mice immunosuppressed with CSA for three months exhibited clonal expansion. The mean clonal abundances of the dominant clone were 42 and 12% in the LMP1/CD40-expressing mice with or without immune suppression, respectively (Student’s *t*-test, *p* = 0.03), without any bias in terms of V segment usage (not shown). These results indicate that long-term CsA-induced immunosuppression significantly favored the expansion of clonal B cells in the LMP1/CD40-expressing mice. Morphologically, the splenic B cells were increased in the mice immunosuppressed with CSA for three months and were associated with broad sheets of large cells with oval nuclei, lacy chromatin and paracentral nucleoli (Fig. 1e). This cell size enlargement was confirmed by flow cytometry, since the forward scatter of the B cells was increased in the immunosuppressed mice (Fig. 1f). Expression of activation markers, such as CD80 and CD86, was upregulated in the IS LMP1/CD40-expressing mice (Fig. 1g). The fraction of BrdU-positive B cells from the immunosuppressed LMP1/CD40-expressing mice was increased compared to that of the controls (Fig. 1h), as were the numbers of Ki67-positive cells in the spleen (not shown). Altogether, these results suggest that long-term immune suppression is associated with both increased clonal expansion and aggressive transformation of indolent LMP1/CD40 B-cell lymphomas.

No significant changes were seen in the blood of the mice after three weeks of T- and NK-cell depletion (not shown). After two months, CsA treatment increased the leukocyte numbers in the LMP1/CD40-expressing mice (Fig. 1i). As assessed by flow cytometry, the B-cell compartment was increased the CsA-treated LMP1/CD40-expressing mice, which was in contrast to the decrease in granulocytes and T cells (Fig. 1j). We also noted the emergence of large B cells on the forward scatter (FCS) monoparametric histograms (Fig. 1k, upper panel). This cell size increase was confirmed by blood smears after May-Grunwald Giemsa (MGG) staining (Fig. 1k, lower panel). Lymphocytes from the control LMP1/CD40-expressing mice remained small, with little cytoplasm, round nuclei and dense chromatin, whereas those from the CsA-treated LMP1/CD40-expressing mice were often large, with an abundant basophilic cytoplasm and prominent nucleoli.

At 5 years, the transformation frequency may be less than 5% for MZLs and greater than 20% for FCLs and CLL.^7^ With the background of a primary genetic event affecting the common precursor tumor cells, such as translocation of BCL2 in FCLs, del(13q) or del(12q) in CLL and MALT1 or BCL10 in MALT MZLs, acquisition of genetic events promoting proliferation and/or resistance to cell death would favor Darwinian selection of more aggressive subclones. This transformation arises either from the dominant clone, such as in most CLL cases,^1, 8^ or from clonally related common precursor tumor cells, such as in FCLs.^8, 9^ Transformation of FL is associated with genetic events favoring immune escape.^9^ In solid cancers, a clear relationship exists between the mutation burden and a positive response to immunotherapies directed against the PD-L1/PD-1 axis.^10^ Acquisition of additional genetic events in indolent lymphomas would be associated with increased immunogenicity of the subclonal B cells. In this context, transformation of an indolent B-cell lymphoma could be due either to aggravation of the patient’s immune deficiency or genetic acquisition of tools able to neutralize the anti-tumor immune response induced by the transformed B-cell while increasing their proliferation index.

Here, we experimentally demonstrated that by removing a control exerted by the immune system, immune suppression of LMP1/CD40-expressing mice was associated with a morphological, immunophenotypic and proliferative shift toward tumor aggressiveness from a preexisting indolent B-cell clone that mimicked the main features of indolent B-cell lymphoma transformation. Thus, as a preclinical model, immunosuppressed LMP1/CD40-expressing mice reproduce the aggressive transformation of an indolent B-cell tumor and highlight the role of immune surveillance during its clinical course. In view of therapies allowing immune restoration, the LMP1/CD40-expressing mouse model opens interesting perspectives not only as a preclinical model of indolent B-cell lymphoma but also as a model of aggressive transformation of a B-cell indolent clone.
